# Long noncoding RNA MALAT1 can serve as a valuable biomarker for prognosis and lymph node metastasis in various cancers: a meta-analysis

**DOI:** 10.1186/s40064-016-3342-7

**Published:** 2016-10-06

**Authors:** Ping Shuai, Yu Zhou, Bo Gong, Zhilin Jiang, Chong Yang, Hongji Yang, Dingding Zhang, Shikai Zhu

**Affiliations:** 1 Sichuan Provincial Key Laboratory for Human Disease Gene Study and Institute of Laboratory Medicine, Hospital of University of Electronic Science and Technology of China and Sichuan Provincial People’s Hospital, Chengdu, 610072 Sichuan People’s Republic of China; 2 Organ Transplant Centre, Hospital of University of Electronic Science and Technology of China and Sichuan Provincial People’s Hospital, Chengdu, 610072 Sichuan People’s Republic of China; 3 Health Management Center, Hospital of University of Electronic Science and Technology of China and Sichuan Provincial People’s Hospital, Chengdu, 610072 Sichuan People’s Republic of China; 4 School of Medicine, University of Electronic Science and Technology of China, Chengdu, 610072 Sichuan People’s Republic of China

**Keywords:** Long noncoding RNA, MALAT1, Cancer, Prognosis, Meta-analysis

## Abstract

**Background:**

Metastasis-associated lung adenocarcinoma transcript 1 (MALAT1), a newly discovered long intergenic noncoding RNA (lincRNA), has been reported to be aberrantly expressed in various cancers, and may serve as a novel potential biomarker for cancer prognosis. This meta-analysis was conducted to investigate the effects of MALAT1 on cancer prognosis and lymph node metastasis.

**Methods:**

A quantitative meta-analysis was performed using a systematic search of PubMed, Medline and Web of Science databases to identify eligible papers on prognostic value of MALAT1 in cancers. The pooled hazard ratios (HRs) or odds ratios (OR) with a 95 % confidence interval (95 % Cl) were calculated to evaluate its prognostic value.

**Results:**

A total of 2094 patients from 17 studies between 2003 and 2016 were included. The results revealed that elevated MALAT1 expression was significantly associated with poor overall survival (OS) in 11 types of cancers (HR = 1.91, 95 % CI 1.49–2.34). Furthermore, subgroup analysis indicated that region of study (Germany, Japan or China), cancer type (digestive system cancers, urinary system cancers or respiratory system cancers) and sample size (more or less than 100) did not alter the significant predictive value of MALAT1 in OS from various types of cancer. In addition, upregulation of MALAT1 expression was significantly associated with poor disease-free survival (HR = 2.29, 95 % CI 1.24–3.35), and recurrence-free survival (HR = 2.09, 95 % CI 0.81–3.37). The results showed that the incidence of lymph node metastasis was higher in high MALAT1 expression group than that in low MALAT1 expression group (OR = 1.67, 95 % CI 1.30–2.15).

**Conclusions:**

This meta-analysis revealed that elevated MALAT1 expression may serve as a novel predictive biomarker for poor survival and lymph node metastasis in different types of cancer.

## Background

Cancer has now become a major cause of morbidity and mortality in most regions worldwide, and the 5-year survival rate remains low in a wide types of cancer (Bray et al. 2013). With the development of genome-wide sequencing technology, numerous investigators are searching for biomarkers that may help with the clinical diagnosis or prognostic prediction of various cancers. Recently, long noncoding RNAs (lncRNAs) have caught increasing attention, and might act as potential valuable biomarkers for cancer diagnosis or potential targets for cancer therapy (Esteller 2011; Gibb et al. 2011).

LncRNAs are generally defined as transcribed RNA molecules with a length greater than 200 nt, and most lack protein coding capability. However, accumulating evidences have suggested that lncRNAs participated in a wide range of biological pathways. LncRNAs could gene expression through diverse molecular mechanisms, including transcriptional and post–transcriptional processing, chromatin modification and epigenetics, genomic imprinting, protein activity modulation and protein localization. Recent studies found that dysregulation of lncRNAs is always associated with cancer development and progression (Zhang et al. 2014; Wang et al. 2014). Importantly, lncRNAs could play important regulators in various cancer-related processes such as modulation of apoptosis and proliferation, drug resistance and the process of epithelial-mesenchymal transition (EMT) (Yang et al. 2014). However, the role of most lncRNAs in cancer progression and prognosis still remains unclear.

Metastasis associated in lung adenocarcinoma transcript 1 (MALAT1) is a highly conserved nuclear-abundant lncRNA with more than 8000 nucleotides derived from chromosome 11q13. It was the first lncRNA that identified as a prognostic biomarker for early stage non-small cell lung cancer (Ji et al. 2003; Guru et al. 1997; van Asseldonk et al. 2000). Up-regulation of MALAT1 expression was reported in a variety of cancers, including non-small cell lung cancer (Ji et al. 2003; Schmidt et al. 2011), hepatocellular carcinoma (Lai et al. 2012), gastric cancer (Okugawa et al. 2014), pancreatic cancer (Liu et al. 2014; Pang et al. 2015), colorectal cancer (Zheng et al. 2014), clear cell renal cell carcinoma (Zhang et al. 2015), esophageal cancer (Hu et al. 2015; Huang et al. 2016a), gallbladder cancer (Wang et al. 2016), bladder cancer (Fan et al. 2014), osteosarcoma (Dong et al. 2015) and breast cancer (Huang et al. 2016b; Miao et al. 2016). MALAT1 is thought to regulate tumor cell viability, invasiveness and migration (Yoshimoto et al. 2016). Recent study found that knock-down of MALAT1 expression could inhibit pancreatic cancer cell proliferation, migration, invasion and the process of epithelial-mesenchymal transition, and induce cell apoptosis and G2/M cell cycle arrest in vitro (Jiao et al. 2014). Moreover, Dong et al. found that that MALAT1 might promote the proliferation and metastasis of osteosarcoma cells by activating the PI3 K/Akt signaling pathway (Dong et al. 2015). Lai et al. demonstrated that downregulation of MALAT1 expression in HepG2 cells suppressed cell viability, motility, and invasiveness and elevated the sensitivity to apoptosis (Lai et al. 2012).

A number of studies have revealed that overexpression of MALAT1 was significantly associated with high-risk grade, metastasis and patients’ survival in many types of cancers (Xia et al. 2016; Wang et al. 2016; Li et al. 2016). Although MALAT1 expression is considered to relating to clinical prognosis of multiple cancers, the specific impact of MALAT1 expression on the outcome of cancer patients still needs to be further investigated. Therefore, we conducted a systematic review and quantitative meta-analysis to further investigate the prognostic role of MALAT1 overexpression in human cancers.

## Methods

### Literature collection

The selected literatures were determined via an electronic search of PubMed, EMBASE, Web of Science, Ovid and Cochrane library databases using these following terms: “MALAT1”, “NEAT2”, “long intergenic noncoding RNA”, “lincRNA”, “lncRNA”, “noncoding RNA”, “cancer”, ‘‘carcinoma”, “neoplasm”, “outcome”, “prognosis”, “prognostic”, “mortality”, “survival”, “metastasis”, “recurrence” and “lymph node metastasis”. The literature search, which was performed by two independent researchers, was limited to the English language. The last search was updated in August 28, 2016. The reference lists of the retrieved articles were searched manually.

### Inclusion and exclusion criteria

The studies were considered eligible if they met the following criteria: any type of human cancer was studied; studies investigating the prognostic role of MALAT1 in various cancers; the levels of MALAT1 expression in cancerous tissues were detected; patients were grouped according to the levels of MALAT1 expression; a link between MALAT1 expression and clinicopathologic parameters was included; studies providing sufficient data to estimate the HRs with corresponding 95 % CI for overall survival (OS), disease specific survival (DSS), disease-free survival (DFS) or disease-free survival (RFS); and studies published in English. Exclusion criteria were as follows: letters, editorials, expert opinions, case reports and reviews; studies only investigated the molecular structure and functions of MALAT1; studies without usable data for further analysis; and duplicate publications.

### Data extraction

Two investigators (PS, YZ) independently evaluated and extracted data from each identified studies based on criteria of inclusion and exclusion. Corresponding authors were contacted to clarify missing or ambiguous data. The following information was carefully extracted: first author, publication date, country, ethnicity, tumor type, sample size, detection method, follow-up period, cut-off value, the number of high and low MALAT1 expression, the number of patients with lymph node metastasis, survival analysis methodology, HRs with corresponding 95 % CIs for OS, DSS, RFS or DFS. HRs with corresponding 95 % CIs were preferentially extracted from univariate or multivariate analyses. If these were not available, the HR estimates were calculated from Kaplan–Meier survival curves using Engauge Digitizer V4.1 (http://sourceforge.net).

### Statistical analyses

All data analyses were performed with STATA statistical software version 14.0 (Stata Corporation, College Station, TX, USA). Pooled HRs with 95 % CIs were used to estimate the strength of the link between MALAT1 and clinical prognosis. *X* ^*2*^-based Cochran Q test and Higgins *I* ^*2*^ statistic were utilized to determine the heterogeneity among the included studies. *P* value <0.05 in combination with *I* ^*2*^ value >50 % was considered significant heterogeneity. Random-effects models were applied in cases with significant heterogeneity. Subgroup analysis and sensitivity analysis were performed to dissect the heterogeneity. In addition, publication bias was determined using Egger’s linear regression test and Begg’s funnel plots analysis. *P*-value <0.05 was considered statistically significant.

## Results

### Studies characteristics

**Fig. 1:**
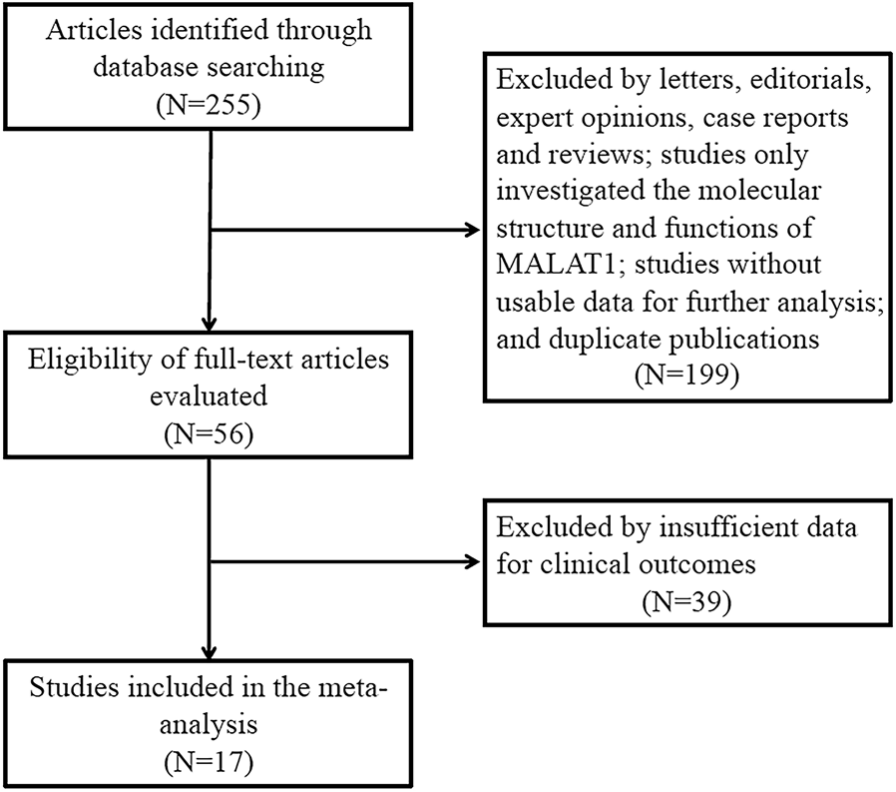
The flow diagram of this meta-analysis

A total of 255 relevant articles were identified based on electronic databases including PubMed, EMBASE, Web of Science, Ovid and Cochrane library. On the basis of the inclusion criteria, 17 eligible papers were enrolled in this meta-analysis (Fig. 1). The characteristics of the included studies are summarized in Table 1. A total of 2094 patients from 17 studies between 2003 and 2016 were included. The regions represented in the studies including Germany (n = 2), Japan (n = 2) and China (n = 13). Among these studies, the sample size ranged between 19 and 352, and 10 studies enrolled more than 100. Eleven types of human cancer were recorded including non-small cell lung cancer (NSCLC), hepatocellular carcinoma (HCC), gastric cancer (GC), pancreatic ductal adenocarcinoma (PDAC), colorectal cancer (CRC), clear cell renal cell carcinoma (ccRCC), esophageal squamous cell carcinoma (ESCC), esophageal cancer (EC), gallbladder cancer (GBC), bladder cancer, osteosarcoma and breast cancer. The levels of MALAT1 expression were detected by reverse transcription quantitative polymerase chain reaction (qRT-PCR) or situ hybridization (ISH). The cut-off values in the included studies were inconsistent partly due to different detection methods. The clinical outcomes were also recoded including 12 studies for OS, 2 for DFS, 2 for PFS, 1 for DSS, and 12 for lymph node metastasis. HRs with the corresponding 95 % CIs were extracted from the original data including 4 studies for OS, 3 for DFS, 1 for DFS, 1 for RFS, and 1 for DFS, and calculated from Kaplan–Meier Curves in 8 studies for OS, 1 for DFS and 1 for RFS.

**Table 1:** The main characteristics of the included studies in the meta-analysis

First author	Year	Region	Ethnicity	Tumor type	Sample size	Cut-off value	Follow-up (months)	Detection method	Survival analysis		Outcome	
Ji P	2003	Germany	Caucasian	NSCLC	225	Median	60 (total)	qRT-PCR	N/A		OS	
Schmidt LH	2011	Germany	Caucasian	NSCLC	352	N/A	N/A	ISH	Univariate		OS	
Lai MC	2012	China	Asian	HCC	164	Mean	36 (total)	qRT-PCR	Univariate	Multivariate	RFS	
Okugawa Y	2014	Japan	Asian	GC	150	ROC	60 (total)	qRT-PCR	Univariate	Multivariate	OS	
Liu JH	2014	China	Asian	PDAC	45	ROC	47 (total)	qRT-PCR	Univariate	Multivariate	DSS	
Zheng HT	2014	China	Asian	CRC	146	Median	60(total)	qRT-PCR	Univariate	Multivariate	OS, DFS	
Zhang HM	2014	China	Asian	ccRCC	106	Mean	60 (total)	qRT-PCR	Univariate	Multivariate	OS	
Fan Y	2014	China	Asian	Bladder cancer	95	Median	30 (total)	qRT-PCR	Multivariate		OS	
Pang EJ	2015	China	Asian	PDAC	126	Median	60 (total)	qRT-PCR	Univariate	Multivariate	OS	
Hu L	2015	China	Asian	EC	54	Mean	N/A	qRT-PCR	N/A		N/A	
Ma KX	2015	China	Asian	Glioma	118	Median	60 (total)	qRT-PCR	Univariate	Multivariate	OS	
Hirata H	2015	Japan	Asian	ccRCC	50	Median	60 (total)	qRT-PCR	Univariate	Multivariate	OS	
Dong YQ	2015	China	Asian	Osteosarcoma	19	Mean	60 (total)	qRT-PCR	N/A		OS	
Huang CX	2016	China	Asian	EC	132	Mean	60 (total)	qRT-PCR	Multivariate		OS	
Huang NS	2016	China	Asian	Breast cancer	204	75 %	65(median)	qRT-PCR	Multivariate		RFS	
Wang SH	2016	China	Asian	GBC	30	Median	40 (total)	qRT-PCR	N/A		OS	
Miao YF	2016	China	Asian	Breast cancer	78	Median	60 (total)	qRT-PCR	N/A		DFS	

*NSCLC* non-small cell lung cancer, *HCC* hepatocellular carcinoma, *GC* gastric cancer, *PDAC* pancreatic ductal adenocarcinoma, *CRC* colorectal cancer, *ccRCC* clear cell renal cell carcinoma, *EC* esophageal cancer, *GBC* gallbladder cancer, *N/A* not available, *qRT-PCR* quantitative real-time PCR, *OS* overall survival, *RFS* recurrence-free survival, *DSS* disease specific survival, *DFS* disease-free survival

### Association between MALAT1 expression and survival in different types of cancers

**Fig. 2:**
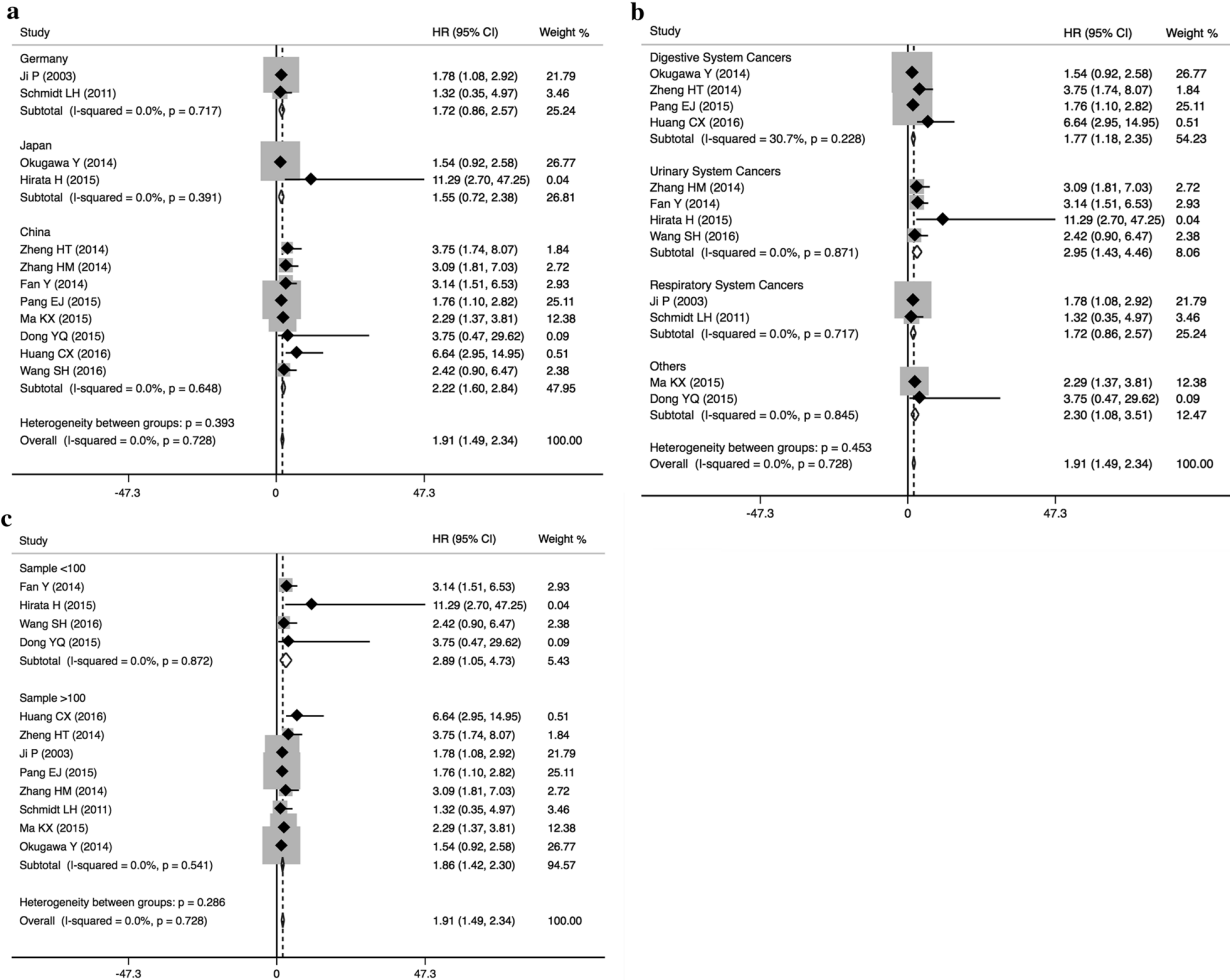
Forest plots of the included studies evaluating the hazard ratios (HRs) for MALAT1 for overall survival (OS). **a** Subgroup analysis of HRs of OS by factor of region; **b** subgroup analysis of HRs of OS by factor of cancer types; **c** subgroup analysis of HRs of OS by factor of sample size

A total of 12 studies including 1549 patients were recruited to assess the effect of MALAT1 overexpression on OS in different types of cancer. It was suggested that elevated MALAT1 expression predicted a poor outcome for OS in 12 types of cancers (pooled HR = 1.91, 95 % CI 1.49–2.34) with no heterogeneity (I^2^ = 0.0 %, *P* = 0.728) (Fig. 2). Furthermore, subgroup analysis was also conducted based on region of study, cancer type and sample size. Stratified analyses by region of study indicated that the different effect of elevated MALAT1 on OS among different countries, including Germany (pooled HR = 1.72, 95 % CI 0.86–2.57), Japan (pooled HR = 1.55, 95 % CI 0.72–2.38), and China (pooled HR = 2.22, 95 % CI 1.60–2.84) (Fig. 2a). A significant relationship was observed between MALAT1 overexpression and poor OS in the studies for digestive system cancers (pooled HR = 1.77, 95 % CI 1.18–2.35), urinary system cancers (pooled HR = 2.95, 95 % CI 1.43–4.46), and respiratory system cancers (pooled HR = 1.72, 95 % CI 0.86–2.57) (Fig. 2b). In addition, the negative effect of MALAT1 overexpression on predicting poor OS was shown in studies including <100 patients (pooled HR = 2.89, 95 % CI 1.05–4.73) as well as those with >100 patients (pooled HR = 1.86, 95 % CI 1.42–2.30) (Fig. 2c).

**Fig. 3:**
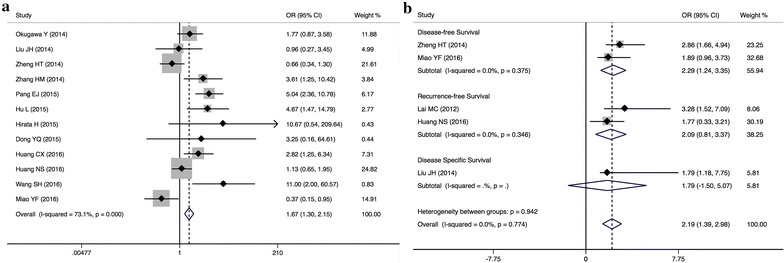
Forest plots of the included studies evaluating (**a**) the odds ratios (ORs) of MALAT1 for lymph node metastasis and (**b**) HRs for MALAT1 expression for DFS, RFS and DSS

In addition, two studies including 224 patients reported HRs for DFS (Fig. 3b). High level of MALAT1 expression was observed to be a negative factor for DFS (pooled HR = 2.29, 95 % CI 1.24–3.35) with no heterogeneity (I^2^ = 0.0 %, *P* = 0.375). Two studies including 368 patients reported HRs for RFS. Upregulation of MALAT1 expression was associated with poor RFS (pooled HR = 2.09, 95 % CI 0.81–3.37) with no heterogeneity (I^2^ = 0.0 %, *P* = 0.346).

### Association between MALAT1 expression and lymph node metastasis

Twelve studies comprising 798 patients were pooled for the meta-analysis of lymph node metastasis. In high MALAT1 expression group, 393 patients (49.2 %) with cancer had lymph node metastasis, while only 405 patients (50.8 %) in low MALAT1 expression group. Due to the presence of heterogeneity (I^2^ = 73.1 %, *P* < 0.001), we compared the incidence of lymph node metastasis between these two groups with the random-effects model. The results found that the incidence of lymph node metastasis was higher in high MALAT1 expression group than that in low MALAT1 expression group (pooled OR = 1.67, 95 % CI 1.30–2.15) (Fig. 3a). This result demonstrated that the patients with high levels of MALAT1 expression are more prone to lymph node metastasis in different systemic cancers.

### Publication bias and sensitivity analysis

**Fig. 4:**
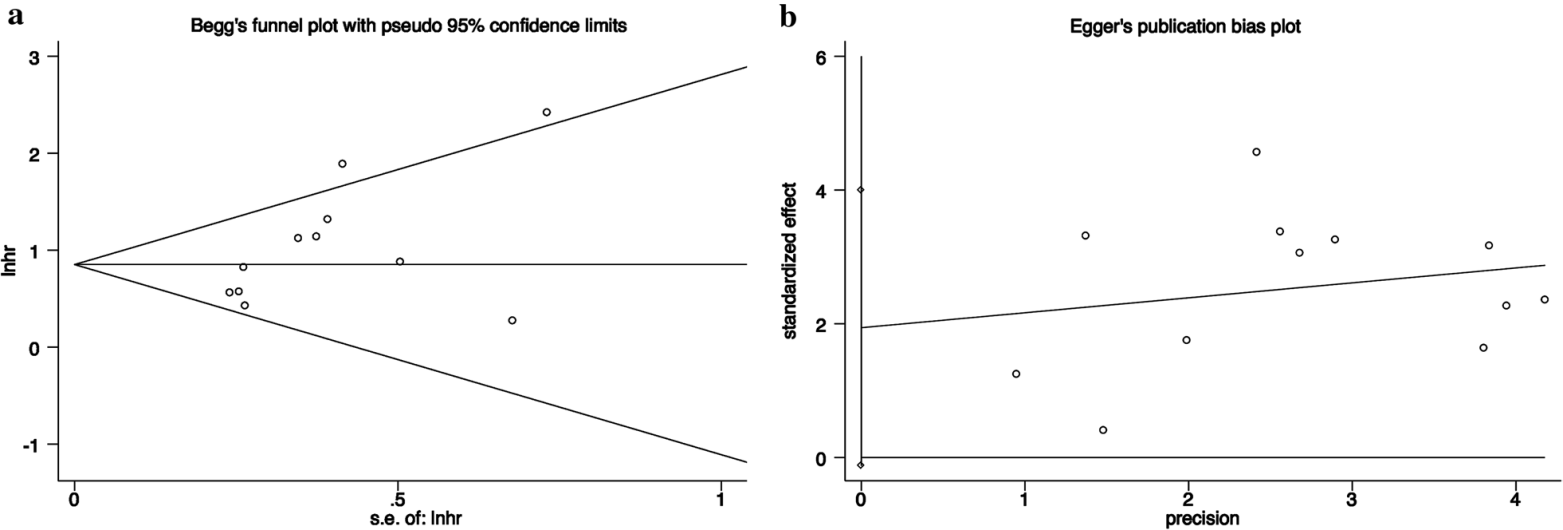
Egger’s linear regression test (**a**) and Begg’s funnel plots analysis (**b**) were performed to evaluate publication bias

To evaluate publication bias in this meta-analysis, the included studies were conducted using Egger’s linear regression test and Begg’s funnel plots analysis. The results of Egger’s linear regression test revealed no publication bias for the analysis of OS (*t* = 2.10, *P* = 0.062). The plots were also asymmetrically inverted funnels (Fig. 4). Due to the small number of studies, publication bias was not analyzed in the DFS and RFS groups. In addition, sensitivity analysis indicated that the pooled HR for deregulated MALAT1 associated with OS was not significantly affected by the exclusion of any of the studies (data not shown).

## Discussion

Although these long noncoding transcripts were once considered to be simply transcriptional noise or cloning artifacts, numerous studies have suggested that lncRNAs are emerging as new players in diverse human diseases, especially cancers (Young and Ponting 2013; Qi and Du 2013). Many studies have demonstrated that lncRNAs are involved in various biological processes, including modulation of apoptosis and proliferation, drug resistance, the process of EMT, cancer progression and metastasis (Hajjari et al. 2014; Yang et al. 2014). An increasing number of studies have focused on the involvement of lncRNAs in cancer development and progression, and proposed them as potential biomarkers for cancer diagnosis or potential targets for cancer therapy (Cui et al. 2016; Yuan et al. 2016; Xie et al. 2016; Liz and Esteller 2016).

MALAT1, regarded as one of the most familiar oncogenic lncRNAs, was overexpressed in various cancers (Yoshimoto et al. 2016). MALAT1 could promote tumor progression through multiple mechanisms in various types of cancer. Increasing reports have demonstrated that MALAT1 could function as a promoter of cell proliferation and metastasis (Thum and Fiedler 2014). Jiao et al. reported that MALAT1 could possibly inhibit G2/M cell cycle arrest, thus promoting EMT in pancreatic cancer (Jiao et al. 2014). Then, Yao et al. found that MALAT1 knockdown induced a decrease in proliferation-enhanced apoptosis, inhibited invasion, and reduced colony formation and led to cell cycle arrest at the G2/M phase in esophageal squamous cell carcinoma (Yao et al. 2016). Xu et al. (2016) revealed that MALAT1 promotes the proliferation of chondrosarcoma cell by activating Notch-1 pathway. Besides, Ji et al. 2013 demonstrated that resveratrol could inhibit invasion and metastasis of colorectal cancer cells though MALAT1 mediated Wnt/beta-catenin pathway . However, the mechanisms underlying aberrant expression of MALAT1 in cancers remain elusive. Some researcher discovered that upregulation of MALAT1 was mediated by the transcription factor Sp1 in A549 lung cancer cells (Li et al. 2015). Additionally, Wang et al. found that silencing of MALAT1 by miR-101 and miR-217 inhibited proliferation, migration, and invasion of esophageal cacner cells (Wang et al. 2015).

Recent studies found that overexpression of MALAT1 correlated with poor prognosis of patients with nearly all types of cancer (Zheng et al. 2014; Zhang et al. 2015; Wang et al. 2016). However, the perplexity and inconsistence arise from a wide range of studies due to heterogeneity. Therefore, a meta-analysis of 2094 cancer patients from 17 studies was undertaken to assess the prognostic value of MALAT1 expression in this study. The pooled data revealed that increased MALAT1 expression was significantly associated with poor OS, DFS and RFS, indicating that MALAT1 has been shown to contribute to cancer progression and considered as a promising prog-nostic biomarker for cancer patients. Furthermore, subgroup analysis indicated that region of study (Germany, Japan or China), cancer type (digestive system cancers, urinary system cancers or respiratory system cancers) and sample size (more or less than 100) did not alter the significant predictive value of MALAT1 in OS from various types of cancer. In addition, MALAT1 was originally discovered to be a marker for metastasis development in early stage lung adenocarcinoma (Ji et al. 2003), and more recently it was shown to be a metastasis-related marker both in squamous cell carcinoma of the lung and hepatocellular carcinoma (Schmidt et al. 2011; Lai et al. 2012). In this present study, we further verified that overexpression of MALAT1 is associated with the incidence of lymph node metastasis in many types of cancer.

Some limitations of this meta-analysis should be acknowledged due to the discrete data across these clinical studies. The major limitation of this meta-analysis was that we only included studies reporting HR or survival curves (except the data that cannot be calculated), and consequently some publications reporting on the prognostic value of MALAT1 were excluded, thus the selection bias might be appeared. Secondly, a part of studies, especially in subgroups analyses, was lightly relative. Thirdly, only summarized data rather than individual patient data were used. Fourthly, some of the HRs could not be directly obtained from the primary studies, we reconstruct the survival curves to extract the HR estimates or calculate them using the reported data. Finally, the cut-off value of high and low MALAT1 expression varied in different studies. Therefore, it is possible that our results might overestimate the prognostic effects of abnormal MALAT1 expression on survival and lymph node metastasis in different types of cancer. We believed that more clinical studies should be conducted to evaluate potential prognostic role of MALAT1 in other types of cancer that have not been included.

## Conclusion

This meta-analysis explored that elevated MALAT1 expression is common to various types of cancer and might act as a novel predictive factor of poor prognosis and lymph node metastasis in different type of cancer. Furthermore, the functional role of MALAT1 in the regulation of cell proliferation, apoptosis and metastasis suggested that MALAT1 may play a key role in tumor progression. Nevertheless, it is still necessary to conduct larger-size and better design studies to confirm our results.
